# The Hope of Immortality

**Published:** 1891-01

**Authors:** Edwin Arnold


### THE HOPE OF IMMORTALITY.

BY EDWIN ARNOLD.

Why, in truth, should evolution proceed along the gross and palpable lines of the visible, and not also be hard at work, upon the subtler elements which are behind—molding, governing and emancipating them ? Taking things as they seem, nobody knows that death stays—nor why it should stay—the development of the individual. It stays our perception of it in another ; but so does distance, absence, or even sleep. Birth gave to each of us much ; death may give very much more, in the way of subtler senses to behold colors we cannot here see, to catch sounds we cannot now hear, and to be aware of bodies and objects impalpable at present to us, but perfectly real, intelligibly constructed, and constituting an organized society and a governed multiform state. Where does nature show signs of breaking off her magic, that she should stop at the five organs and the sixty odd elements ? Are we free to spread over the face of this little earth, and never free to spread through the solar system and beyond it? Nay, the heavenly bodies are to the ether which contains them, as mere spores of seaweed floating in the ocean. Are the specks only filled with life, and not the space ? What does nature possess more valuable in all she has wrought here, than the wisdom of the sage, the tenderness of the mother, the devotion of the lover, and the opulent imagination of the poet, that she should let these priceless things be utterly lost by a quinsy or a flux. It is a hundred times more reasonable to believe that she commences afresh with such delicately developed treasures, making them groundwork and stuff for splendid farther living, by process of death ; which, even when it seems accidental or premature, is probably as natural and gentle as birth ; and wherefrom, it may well be, the new born dead arises to find a fresh world ready for his pleasant and novel body, with gracious and willing kindred ministrations awaiting it, like those which provided for the human babe the guiding arms and nourishing breasts of its mother. As the babe's eyes opened to strange sunlight here, so may the eyes of the dead lift glad lids to "a light that never was on sea or land ;" and so may his delighted ears hear speech and music proper to the spheres beyond, while he laughs contentedly to find how touch and taste and smell had all been forecasts of faculties accurately following upon the lowly lessons of this earthly nursery.

